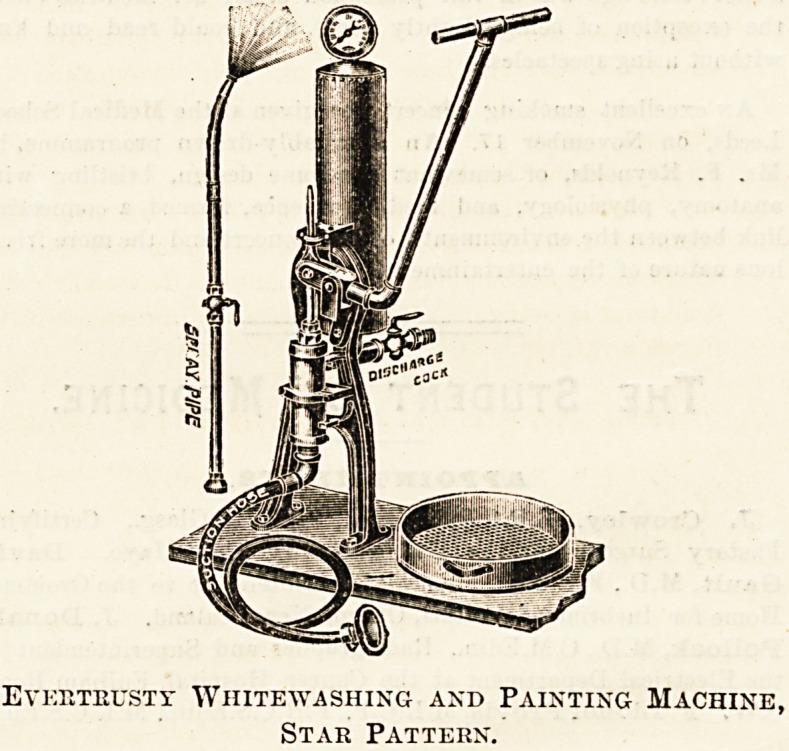# Practical Departments

**Published:** 1903-12-05

**Authors:** 


### PRACTICAL DEPARTMENTS.

### AN ADAPTABLE BATH CHAIR.

THE illustration below shows an adaptation of an ordinary invalid's chair to the purposes of a couch, sent us by

Miss M. Horne, of Castle Grounds School, Devizes, who has obtained a provisional protection for her contrivance. It is a strong linen hammock suspended from handle-bar to support forming foot-bar of wicker (or other) bath chair, forming an open-air couch and a convertible spinal carriage, or carriage for nervous and heart cases. It is perfectly simple and easy to manipulate. The expense is small compared with ordinary convertible invalid carriages. The result obtained is perfect freedom from vibration and ease of position. The occupant swings quite clear of the chair, and can be pulled up into reclining position without being disturbed. Mr. Llewellin, King Square, Bristol, patent expert, is authorised to negotiate with firms wishing to manufacture on license or sell on royalty. It lends itself admirably to the "open-air cure" treatment.

### A WHITEWASHING AND PAINTING MACHINE.

THE accompanying illustration shows a simply-constructed invention whereby lime and paint can be applied to walls, floors, etc., in less time and at much less cost than is the case when the ordinary means are employed—viz, the brush and pot. The machine is, in fact, a pump; the air, compressed in the upright cylinder, forcing the liquid into

EVERTRUSTY WHITEWASHING AND PAINTING MACHINE, STAR PATTERN.

the hose from a tank or pail connected by a short length tube. Two workers are required, one to pump and one to guide the hose. This is fitted with a spray nozzle bent so that the liquid strikes the wall at right angles. We have seen the machine at work with water only, and are assured by the makers that the pipes, etc., do not corrode or get clogged when lime is used, all the working parts being of brass, copper, bronze, and steel, but that lime, whitewash, distemper, and cold-water paints must be strained before use. They state that an even surface is not difficult to obtain, and that interstices in a rough wall may be filled in with ease. Although its primary use is for the application of white or colour wash, the "Star" machine is also used for spraying disinfectants (for this purpose we understand it is in use in the plague districts in India), and for cleaning walls, ceilings, floors, etc., as well as for fire-extinguishing purposes.

The saving to hospitals and institutions by using this simple machine should obviously be worth consideration, since skilled labour is unnecessary, and the work is such that it could easily be performed by convalescents, for whom light occupation is considered desirable. A boy can work the pump, and from 50 to 60 gallons can be passed through in an hour. The working capacity of the machine we inspected, standing about 3 feet high, is stated to be equal to that of 25 men with brushes. A small machine is made at a trifling cost, with a working capacity equal to five men; this would probably be sufficiently powerful for cottage hospital use.

A cold-water paint called Pnumapant is supplied by the firm to replace white and lime wash, and it is stated that the covering capacity of this is much greater than that of any other preparation *e.g.*, one gallon will cover from 50 to 75 square feet of smooth unpainted boards, or 25 to 40 square feet of rough unpainted boards and stone.

The proprietors are Messrs. Wallach Bros., 57 Gracechurch Street, London, E.C., and 108A Hope Street, Glasgow.

## Figures and Tables

**Figure f1:**
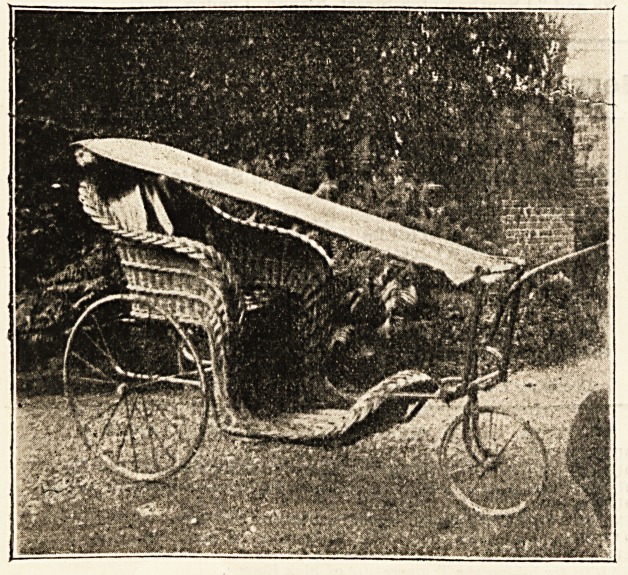


**Figure f2:**